# 

**Published:** 1842-07

**Authors:** 


### WESTERN JOURNAL

OF

### MEDICINE AND SURGERY:

EDITED BY

DANIEL DRAKE, M. D.

AND

LUNSFORD P. YANDELL, M. D.

PROFESSORS IN THE LOUISVILLE MEDICAL INSTITUTE,

AND

THOMAS W. COLESCOTT, M. D.

---

NO. XXXI.—JULY, 1842.

Vol. VI.—No. I.

---

LOUISVILLE, KY.

PUBLISHED MONTHLY, BY PRENTICE & WEISSINGER,

PROPRIETORS AND PRINTERS.

1842.

*This Number contains* 4 *sheets—Postage under* 100 *miles* 6 *cents, over* 100 *miles* 10 *cents.*

